# Associations between midlife chronic conditions and medication use with anxiety and depression: A cross-sectional analysis of the PREVENT Dementia study

**DOI:** 10.1177/2235042X20920443

**Published:** 2020-05-05

**Authors:** Lucy E Stirland, Sarah Gregory, Tom C Russ, Craig W Ritchie, Graciela Muniz-Terrera

**Affiliations:** 1 Edinburgh Dementia Prevention, Centre for Clinical Brain Sciences, University of Edinburgh, Edinburgh, UK; 2 Division of Psychiatry, Centre for Clinical Brain Sciences, University of Edinburgh, Edinburgh, UK; 3 Alzheimer Scotland Dementia Research Centre, University of Edinburgh, Edinburgh, UK; 4 Psychiatry of Old Age, NHS Lothian, Edinburgh, UK

**Keywords:** Multimorbidity, polypharmacy, depression, middle-aged, anxiety disorders

## Abstract

**Background:**

Multimorbidity including physical and mental illness is increasing in prevalence. We aimed to investigate the associations between physical conditions and medication use with anxiety and depression in midlife.

**Methods:**

We conducted an observational cross-sectional study of volunteers in the PREVENT Dementia study. Using logistic and linear regression, we investigated the association between increasing numbers of self-reported chronic physical conditions and medications with self-reported depression and anxiety disorder, and scores on the Center for Epidemiologic Studies Depression (CES-D) scale and Spielberger State-Trait Anxiety Inventory (STAI) state subtest.

**Results:**

Of the 210 participants, 148 (71%) were women and 188 (90%) Caucasian. The mean age was 52 (standard deviation (SD) = 5.5) years. The mean number of physical conditions was 2.2 (SD = 1.9) and medications 1.7 (SD = 2.2). Each additional physical condition was associated with increased odds of self-reported depression (odds ratio (OR) 1.41, 95% confidence interval (CI) 1.11–1.80; *p* = 0.004, adjusted for age and gender) and anxiety disorder (OR 1.70, 95% CI 1.30–2.37; *p <* 0.001). Increasing medication use was associated with self-reported depression (adjusted OR per additional medication 1.35, 95% CI 1.08–1.71; *p* = 0.008) but not anxiety disorder. For each additional condition, CES-D scores increased by 0.72 (95% CI 0.11–1.33; *p* = 0.020) and for each extra medication, by 0.88 (95% CI 0.32–1.44; *p* = 0.002). There was no significant association between increasing conditions and medications with STAI scores. In models accounting for antidepressant use, all associations were attenuated.

**Conclusions:**

Having more physical conditions is associated with anxiety and depression in midlife, and taking more medications is associated with depression but not anxiety.

## Introduction

Multimorbidity (the coexistence of multiple chronic diseases) and polypharmacy (taking many different medications) are complex areas attracting increasing research and policy attention.^1^ Although often linked with older age, multimorbidity and polypharmacy are becoming more prevalent in midlife.^2,3^ Existing research shows that multimorbidity including both physical and mental illness is common and associations between physical and mental health are likely to be bidirectional.^2,4^

The evidence regarding the interplay between multimorbidity and depression or anxiety in midlife is limited. For example, regarding depression, a recent systematic review revealed that adults with multimorbidity had a three times greater risk of depression than people with no chronic physical conditions.^5^ However, only one of the 40 studies included in that review investigated a midlife cohort (aged 45–64 years), with 26 studies on older people and 13 on adults of all ages, reflecting the fact that research on multimorbidity and polypharmacy tends to focus on older age groups.^6^ With regard to anxiety, cross-sectional studies have shown that multimorbidity and anxiety coexist.^7,8^ There is some evidence that increasing numbers of medication are associated with more depressive symptoms, but this area is less well understood.^9^ Furthermore, there is little research into associations between polypharmacy and anxiety.

Midlife cohorts are increasingly studied in relation to the early manifestations of neurodegenerative diseases that may later lead to dementia. Identifying risk–disease interactions may contribute to reducing incidence via risk modification strategies.^10^ Depression and anxiety in midlife have been identified as risk factors for dementia,^11,12^ although the direction of the association remains uncertain.^13^ Therefore, understanding the interplay between multimorbidity and polypharmacy with depression and anxiety is crucial, given the fact that all four have been associated with poor brain health and dementia.

PREVENT Dementia is an ongoing cohort study designed to investigate midlife risk factors for neurodegenerative diseases.^14^ It offers opportunities to explore the associations between multimorbidity, polypharmacy, depression and anxiety in midlife and to allow better understanding both of this age group and of future brain health.

## Objective

We aimed to investigate whether increasing numbers of chronic conditions and medications were associated with depression and anxiety in this cross-sectional midlife cohort.

## Methods

### Participants

This is an observational cross-sectional study of a convenience sample of volunteers in the first phase of PREVENT Dementia (a dementia prevention study) in London, UK. Volunteers were eligible to participate in PREVENT Dementia if they were aged 40–59 years at baseline and were fluent in English. Potential participants who reported having cognitive impairment or dementia were excluded, as were those with known contraindication to magnetic resonance imaging (MRI). Recruitment took place through a local database (DemReg),^15^ the UK-wide Join Dementia Research database (JDR),^16^ via publicity online and at public presentations. The DemReg and JDR databases are both open to anyone aged 18 years and over who consent to be contacted about research. Recruitment to DemReg was facilitated via memory clinics, meaning those in the age group of interest for this study were likely attending the clinic as a family member of a patient. JDR is an online database and, therefore, is available to anyone with Internet access. These databases were selected as the major recruitment tools for the study as they contained contact details of volunteers meeting inclusion criteria who were motivated to participate in research studies. The study team aimed to recruit half of the participants with a family history of dementia and half without. All participants gave written informed consent and approval for the study was given by the NHS Research Ethics Committee, Camberwell St Giles. Participants underwent in-depth physical and cognitive testing, comprehensive medical, lifestyle and mental health questionnaires, brain MRI and fMRI and provided neurodegenerative disease biomarkers.^17^ The study protocol is published in detail elsewhere, including justification of the predefined minimum sample size of 150 participants.^14^

### Depression measures

Depressive symptoms were measured using the Center for Epidemiologic Studies Depression (CES-D) scale.^18^ The CES-D is a validated self-administered scale containing 20 questions about depressive symptoms and scored out of 60. The questions cover mood, cognitive and somatic symptoms of depressive disorder, and participants rate how often they have experienced them in the past week (0 = *less than 1 day*, 1 = *1–2 days*, 2 = *3–4 days* and 3 = *5–7 days*). Although a cut-off of ≥16 is generally used to identify people with depression, the participant’s rating for each item measures frequency of each reported symptom, so any increase may be of clinical interest.^19^ In addition, even low levels of psychological distress have been associated with negative outcomes including mortality.^20^ We anticipated that only a small proportion of this cohort of volunteers would be classified as depressed so chose to analyse raw scores as a pseudo-continuous variable. Participants’ self-report of an active, current diagnosis of depression came from the medical history, which was taken at interview by a qualified doctor.

### Anxiety measures

Anxiety symptoms were measured using the Spielberger State and Trait Anxiety Inventory (STAI) state subtest.^21^ It consists of 20 questions on symptoms of anxiety, scored from one to four based on participants’ reported severity (*not at all*, *somewhat*, *moderately so* and *very much so*) resulting in a score between 20 and 80. A cut-off of ≥40 for clinically significant anxiety is frequently used, although higher cut-offs have been shown to have higher accuracy at detecting clinical anxiety disorders in older people.^22^ As the STAI was originally designed as a continuous scale and we were interested in symptoms, we again used the overall score as a pseudo-continuous variable. Participants’ self-report of current anxiety disorder was taken from the medical history.

### Chronic conditions

The PREVENT Dementia case report medical history includes a list of medical conditions. Participants were asked whether they had ever had each condition and whether it was currently active. They also had the opportunity to report other conditions, which were recorded as free text by the interviewing doctor. We reviewed all potential conditions and defined them as chronic if they were likely to be present for at least six months, have an impact on quality of life and have a pattern of recurrence or deterioration. This definition was based on a combination of definitions from the International Classification of Primary Care, version 2 and from the NHS National Services Scotland Information Services Division.^23,24^ Depending on the nature of each condition, some were included if they had ever been diagnosed and others only if they were active. We excluded psychiatric disorders due to their overlap with our outcomes. This left 55 possible chronic physical conditions, which are listed, with their duration definitions, in Appendix 1. Multimorbidity is commonly defined as the coexistence of two or more conditions and many studies use dichotomous variables (e.g. 0–1 versus 2 conditions). However, this approach does not capture the full distribution of conditions, particularly at the higher extremes.^25^ We, therefore, used continuous counts of conditions as exposure variables for analyses.

### Medication history

At the research interview, study doctors collected information on current medication use according to participant self-report. This included drug name, dose, frequency and indication. The reported medications were then coded according to the World Health Organization’s (WHO) Anatomical Therapeutic Chemical (ATC) classification system.^26^ Over-the-counter vitamins or health supplements were excluded, as were entries with insufficient information to generate an ATC code. Due to the likely effect of antidepressant use on both depression and anxiety outcomes, we excluded antidepressants from the total count of medications. We aimed to account for anxiolytic medications but included them in the overall count. We used this adjusted medication count as an exposure variable in regression models.

### Additional variables

Participants reported their age and gender, which we included in all regression models as these are clinically relevant factors influencing depression and anxiety symptoms. Use of antidepressants was both clinically relevant and statistically significant in preparatory analyses. Considering that antidepressants are used for several indications, we reviewed the free text records on medication indication and generated a variable for antidepressant use for any psychiatric indication. We included this variable in a separate adjusted model and tested for interaction effects between chronic conditions and antidepressant use. We conducted sensitivity analyses in a sample excluding participants who took antidepressants for psychiatric indications. We also created a variable for using ATC-coded anxiolytic medications.

### Statistical analysis

All analyses were run in R version 3.4.3.^27^ We used Student’s *t*-test to compare the mean age, chronic conditions and medications between people with and without self-reported depression and anxiety disorder. Linear regression models were used for the continuous outcome variables (CES-D and STAI scores) and logistic regression for binary outcomes, namely, the presence of self-reported depression and anxiety disorder. Owing to the disproportionate gender split, we performed additional analyses stratified by gender.

## Results

### Description of the sample

The sample, from the pilot phase of PREVENT Dementia in London, UK, consisted of 210 individuals, 148 (70.5%) of whom were women. The mean age was 52.0 (SD = 5.5) years and median 53 years. Self-reported race was Caucasian for 89.5% of participants with the next largest groups being Black (*n* = 7, 3.3%) and Indian subcontinent (*n* = 7, 3.3%). Almost half (103, 49.0%) of the participants had a first-degree relative with dementia; 10 (4.8%) were current smokers, 80 (38.1%) were ex-smokers and 120 (57.1%) had never smoked. The mean weekly alcohol intake was 11.5 units (SD = 12.4), and the mean body mass index was 27.7 kg/m^2^ (SD = 5.3). The principal demographic details are listed in Table 1. The mean number of chronic physical conditions was 2.2 (SD = 1.9), with a range of 0–9. The mean number of medications reported was 1.7 (SD = 2.2) and range 0–12. After excluding antidepressants, the mean number of medications was 1.5 (SD = 2.0). Only one participant (0.5%) was taking an anxiolytic medication so due to low prevalence, this variable was not included in further analyses. Appendix 1 lists all the included conditions with their prevalence in this sample. There were no missing data for any of the variables included.

**Table 1.** Sample characteristics in whole sample (*n* = 210).

Variable	*n* (%)	Mean (SD)
Gender (female)	148 (70.5)	
Race (Caucasian)	188 (89.5)	
Current depression (self-report)	16 (7.6)	
Current anxiety disorder (self-report)	21 (10.0)	
Taking antidepressant for any indication	26 (12.4)	
Taking anxiolytic medication	1 (0.5)	
Age (years)		52.0 (5.5)
Education (years)		15.9 (3.4)
CES-D Total (possible range 0–60)		9.2 (8.2)
STAI Total (possible range 20–80)		30.4 (9.4)
Number of chronic physical conditions		2.2 (1.9)
Number of current medications including antidepressants		1.7 (2.2)
Number of current medications excluding antidepressants		1.5 (2.0)

SD: standard deviation; CES-D: Center for Epidemiologic Studies Depression; STAI: State-Trait Anxiety Inventory.

**Figure 1.**
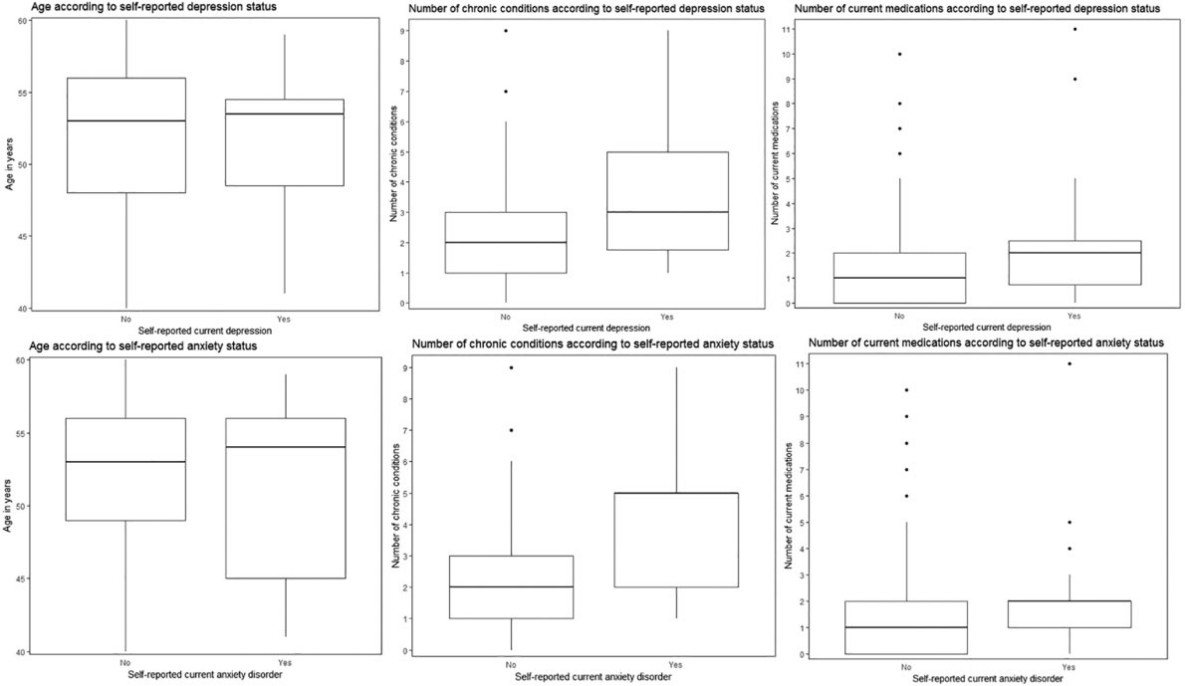
Box plots of age, chronic conditions and medication for self-reported outcomes in whole sample (*n* = 210).

For participants with at least one chronic condition, the mean number of medications per condition was 0.7 (SD 0.9). Among all participants, 119 (56.7%) had two or more conditions and 48 (22.9%) people took three or more medications (39 (18.6%) excluding antidepressants). There was a statistically significant difference between the mean number of chronic physical conditions among people with and without self-reported depression (*µ*_1_ = 3.6, *µ*_2_ = 2.1; *p* = 0.025) but not number of medications or age (Table 2). For people with and without self-reported anxiety disorder, there was a difference in the mean number of chronic conditions (*µ*_1_ = 4.2, *µ*_2_ = 2.0; *p* < 0.001) but not the number of medications or age. Figure 1 shows box plots of these distributions.

**Table 2.** Characteristics of participants reporting depression or anxiety disorder.

	No depression	Self-reported depression	*p* for difference (Student’s *t*-test)	No anxiety disorder	Self-reported anxiety disorder	*p* for difference (Student’s *t*-test)
Mean age in years (SD)	52.0 (5.4)	51.3 (6.3)	0.642	52.0 (5.4)	51.4 (6.3)	0.656
Mean number of chronic physical conditions (SD)	2.1 (1.8)	3.6 (2.5)	0.025	2.0 (1.6)	4.2 (2.4)	<0.001
Mean number of medications taken (excluding antidepressants) (SD)	1.4 (1.8)	2.7 (3.2)	0.140	1.4 (1.9)	2.2 (2.5)	0.174

SD: standard deviation; CES-D: Center for Epidemiologic Studies Depression; STAI: State-Trait Anxiety Inventory.

We found that 26 (12.4%) participants were taking antidepressants, of whom 18 (8.6%) were doing so for psychiatric indications. Within this group, 13 (72.2%) participants reported a diagnosis of depression and 12 (66.7%) reported anxiety disorder. We tested for interaction effects between chronic conditions and antidepressant use and found no statistically significant interaction. Table 3 presents the characteristics of participants according to their antidepressant status; there was a significantly higher rate of self-reported depression and anxiety disorder among those taking antidepressants for a psychiatric indication. People taking antidepressants also had significantly higher mean CES-D scores (*µ*_1_ = 14.3, *µ*_2_ = 8.7; *p* = 0.021) and mean chronic conditions (*µ*_1_ = 3.7, *µ*_2_ = 1.4; *p* = 0.006).

**Table 3.** Characteristics of sample based on antidepressant status.

	Not taking antidepressant for psychiatric indication (*n* = 192)		Taking antidepressant for psychiatric indication (*n* = 18)		
Variable	*n* (%)	Mean (SD)	*n* (%)	Mean (SD)	*p* for difference (Student’s *t*-test or χ^2^ test)
Gender (female)	132 (68.8)		16 (88.9)		0.128
Race (Caucasian)	172 (89.6)		16 (88.9)		1
Current depression (self-report)	3 (1.6)		13 (72.2)		**<0.001**
Current anxiety disorder (self-report)	9 (4.7)		12 (66.7)		**<0.001**
Age (years)		51.9 (5.5)		52.1 (5.8)	0.907
Education (years)		15.9 (3.3)		16.5 (4.6)	0.575
CES-D Total (possible range 0–60)		8.7 (7.9)		14.3 (9.1)	**0.021**
STAI Total (possible range 20–80)		30.0 (9.4)		34.0 (9.1)	0.090
Number of chronic physical conditions		2.0 (1.8)		3.7 (2.2)	**0.006**
Number of current medications excluding antidepressants		1.4 (1.8)		2.9 (3.)	0.052

SD: standard deviation; CES-D: Center for Epidemiologic Studies Depression; STAI: State-Trait Anxiety Inventory.

Bold figure indicates *p* < 0.05.

### Depression outcomes

On the CES-D, 35 (16.7%) participants scored 16 or over, which is the accepted cut-off for depression. The mean CES-D score was 9.2 (SD = 8.1). Sixteen people (7.6%) reported a diagnosis of depression in their medical history, and of these, seven (44.0%) scored above the 16 cut-off point on the CES-D. Nine (4.3%) participants reported both depression and an anxiety disorder.

With each additional physical condition, the CES-D score increased by 0.72 units (95% CI 0.11–1.33; *p* = 0.020) after adjustment for age and gender. However, the estimate dropped below conventional significance levels when we additionally adjusted for antidepressant use (*β* = 0.56, 95% CI −0.06–1.18; *p* = 0.078) and in a subsample of participants who did not take antidepressants for psychiatric indications (*β* = 0.43, 95% CI −0.23–1.08; *p* = 0.199).

Similarly, although each additional medication emerged as associated with higher CES-D scores (*β* = 0.88, 95% CI 0.32–1.44; *p* = 0.002, adjusted for age and gender), even when including antidepressant use as a covariate (*β* = 0.74, 95% CI 0.18–1.31; *p* = 0.011), testing the association in a subsample of those not taking antidepressants rendered it non-significant (*β* = 0.53, 95% CI −0.09–1.16; *p* = 0.094).

The odds ratio (OR) for self-reported depression with the number of chronic physical conditions, adjusted for age and gender, was 1.41 (95% CI 1.11–1.80; *p* = 0.004). Additionally adjusting for antidepressant use reduced the OR to 1.26 (0.83–1.90; *p* = 0.273). The OR adjusted for age and gender per unit increase in number of medications for self-reported depression was 1.35 (1.08–1.71; *p =* 0.008). This OR reduced to 1.13 (0.79–1.70; *p* = 0.545) when additionally adjusting for antidepressant use with psychiatric indication.

Both increasing number of medications and increasing chronic conditions were associated with increasing CES-D score and self-reported depression. These associations were attenuated when accounting for antidepressant use for psychiatric indications and became no longer statistically significant at conventional levels. All regression analysis results are summarized in Table 4.

**Table 4.** Summary of regression analysis results.

		Exposure					
Outcome	Model	Chronic physical conditions			Medications excluding antidepressants		
		Coefficient (95% CI)	OR^a^ (95% CI)	*p* Value	Coefficient (95% CI)	OR^a^ (95% CI)	*p* Value
Depression							
CES-D	Model 1	**0.72 (0.11, 1.33)**		**0.020**	**0.88 (0.32, 1.44)**		**0.002**
	Model 2	0.56 (−0.06, 1.18)		0.078	**0.74 (0.18, 1.31)**		**0.011**
	Model 3^b^	0.43 (−0.23, 1.08)		0.199	0.53 (−0.09, 1.16)		0.094
Self-reported depression	Model 1		**1.41 (1.11, 1.80)**	**0.004**		**1.35 (1.08, 1.71)**	**0.008**
	Model 2		1.26 (0.83, 1.90)	0.273		1.13 (0.79, 1.70)	0.545
	Model 3^b^		NA			NA	
Anxiety							
STAI	Model 1	0.14 (−0.57, 0.85)		0.704	0.27 (−0.39, 0.92)		0.425
	Model 2	0.01 (−0.72, 0.73)		0.986	0.16 (−0.51, 0.83)		0.637
	Model 3^b^	0.06 (−0.71, 0.84)		0.871	0.20 (−0.54, 0.95)		0.588
Self-reported anxiety disorder	Model 1		**1.70 (1.35, 2.19)**	**<0.001**		1.23 (0.99, 1.51)	0.045
	Model 2		**1.73 (1.30, 2.37)**	**<0.001**		1.04 (0.78, 1.36)	0.800
	Model 3^b^		NA			NA	

OR: odds ratio; CI: confidence interval; CES-D: Center for Epidemiologic Studies Depression; STAI: State-Trait Anxiety Inventory.

^a^ OR per unit increase in number of chronic conditions or medications.

^b^ N.B. smaller sample size, as below: model 1: whole sample (*n* = 210), adjusted for age and gender; model 2: whole sample (*n* = 210), adjusted for age, gender and use of antidepressants for psychiatric indication; model 3: sample excluding participants taking antidepressants for psychiatric indication (*n* = 192), adjusted for age and gender (not calculated for self-reported diagnoses of depression and anxiety disorder due to high proportion of people with diagnoses taking medication).

Bold figure indicates *p* < 0.05.

### Anxiety outcomes

The mean score on the STAI was 30.4 (SD = 9.4). Twenty-one participants (10%) reported a diagnosis of anxiety disorder in their medical history; of these, 7 (33.3%) scored above the cut-off of 40 on the STAI and 18 had ≥2 physical conditions.

There were no significant associations between an increasing number of chronic conditions and the STAI state score in a model adjusted for age and gender (*β* = 0.14, 95% CI −0.57–0.85; *p* = 0.704). This remained non-significant when additionally adjusting for antidepressant use (*β* = 0.01, 95% CI −0.72–0.73; *p* = 0.986). The regression coefficient for the effect of each additional medication on the STAI score was *β* = 0.27 (95% CI −0.39–0.92; *p* = 0.425) and this remained non-significant when adding antidepressant use as a covariate (*β* = 0.16, 95% CI −0.51–0.83; *p* = 0.637). In the subsample of participants who did not take antidepressants for a psychiatric indication, the associations between both chronic conditions and medication with STAI did not meet conventional significance levels (presented as model 3 in Table 4).

The OR (95% CI) adjusted for age and gender for self-reported anxiety disorder with number of chronic conditions was 1.70 (1.35–2.19; *p* < 0.001). Additionally adjusting for antidepressant use increased the OR to 1.73 (1.30–2.37; *p* < 0.001). The OR (95% CI) adjusted for age and gender per unit increase in number of medications for self-reported anxiety disorder was 1.23 (0.99–1.51; *p* = 0.045). This OR remained non-significant at 1.04 (0.78–1.36; *p* = 0.800) when additionally adjusting for antidepressant use.

### Analyses stratified by gender

The results of regression analyses stratified by gender are presented in Appendix 2. Table 2A shows that in women, there were associations between chronic physical conditions and medications with CES-D scores and self-reported depression. There were also associations between conditions, but not medications, and self-reported anxiety disorder. Additionally adjusting for antidepressant use rendered the associations non-significant, apart from the model including chronic conditions and self-reported anxiety disorder. By contrast, in men, the only significant association was between increasing medication use and increasing CES-D scores. Depression was reported by one (1.6%) male participant and anxiety disorder by two (3.2%) male participants, so we did not conduct analyses with self-reported depression or anxiety disorder as outcomes in men.

**Figure 2.**
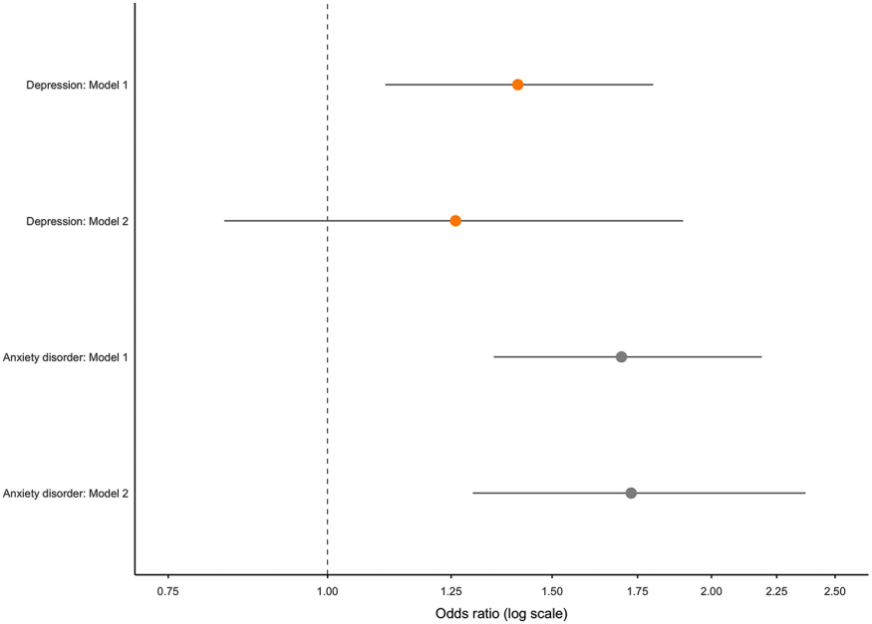
OR (95% CI) of self-reported outcomes with each additional chronic physical condition: whole sample (*n* = 210). OR: odds ratio; CI: confidence interval.

**Figure 3.**
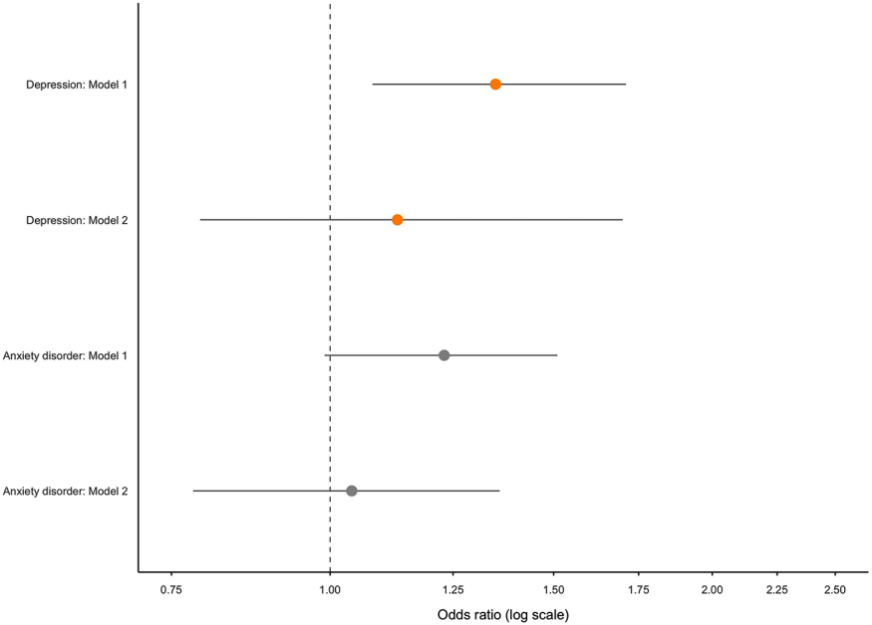
OR (95% CI) of self-reported outcomes with each additional medication: whole sample (*n* = 210). OR: odds ratio; CI: confidence interval.

Figures 2 and 3 show the OR and 95% CI for self-reported depression and anxiety per unit increase in chronic conditions and medications, respectively.

In summary, no clear association between increasing medication use and anxiety measures emerged. Chronic conditions were associated with self-reported anxiety disorder but not increasing STAI scores.

## Discussion

### Key results

We found associations between increasing chronic conditions and self-reported depression, increasing CES-D scores and self-reported anxiety disorder, but not STAI score. There were associations between increasing numbers of medication with both self-reported depression and increasing CES-D scores. There was no association between increasing medication count and anxiety, either self-reported or according to the STAI. The findings no longer met conventional significance levels when adjusting for antidepressant use, suggesting that a preexisting diagnosis with partial treatment may explain the observed associations.

### Comparison to existing literature

Participants in the initial wave of the PREVENT Dementia study had a mean of 2.2 chronic physical conditions. Recent publications in similar age groups found figures of 0.8 chronic conditions in an English primary care cohort and 1.2 chronic conditions in Scottish data.^2,28^ The apparently above-average prevalence of multimorbidity in PREVENT Dementia participants may reflect the self-report method of gathering medical history.

The majority of PREVENT Dementia participants were taking at least one medication and among those, the mean number of medications taken was 2.6 (SD 2.2). In contrast, a population-level analysis in one region of Scotland found that among adults (mean age 50.1 years) prescribed any medication, the mean was 4.4 prescribed medications.^29^ The PREVENT Dementia cohort reported more than would be expected in terms of medical conditions but were receiving less than would be expected in terms of medication. This could imply a population that is very observant of their own health but reporting conditions not severe enough to require treatment. This discrepancy is, therefore, likely due to the use of volunteers in PREVENT Dementia.

It is difficult to compare multimorbidity studies when there is disparity between the number of possible conditions listed in each of them. Previous studies reviewing prevalence estimates of multimorbidity using disease counts have recommended using a list of at least 12 conditions^30^ and between 25 and 75 conditions.^31^ Our list of 55 conditions is likely to have been more sensitive than those in previous similar studies, with the consequent risk of over-estimation of multimorbidity.

We found an association between increasing number of medications (excluding antidepressants) and scores on the CES-D and self-reported depression. The only similar study using this measure was published in 1989 and found a similar association but did not account for antidepressant use.^9^ There has also been an increase in the prevalence of polypharmacy since then.^3^ There is little in the literature about medication use and specific anxiety outcomes, so our analyses are novel in this area.

A systematic review and meta-analysis of 40 articles found an OR of 1.45 (95% CI 1.28–1.64; *p* < 0.001) for depression with each additional condition, which our analyses of self-reported depression support. All of the articles reviewed used either a depression rating scale or clinical diagnosis; none considered both.^5^ A very large cross-sectional study of primary care patients with depression and controls found that people with depression were more likely to have multimorbidity and that this association was stronger in people with socioeconomic deprivation.^32^ This was a representative sample from primary care, but used routinely collected diagnostic information from health records and symptom measures were not available. Our research builds on this evidence by assessing both self-reported diagnoses and symptom scales as outcomes.

There have been two cross-sectional studies specifically exploring the link between multimorbidity and anxiety, both of which found statistically significant associations. One was a large international study of adults aged over 18 years, which measured multimorbidity from a list of nine conditions and anxiety with a single question answered on a 5-point scale.^7^ The other measured anxiety according to the Beck Anxiety Inventory and multimorbidity from a list of seven conditions, in participants aged over 65 years.^8^ Again, there is strength in our research using both self-reported diagnosis and symptom scales; we found a similar association between increasing chronic conditions and anxiety disorder but not symptoms as reported on STAI. This difference between reported diagnosis and objective measurement may reflect the fact that those who report a diagnosis are likely to be receiving treatment and, therefore, report fewer active symptoms.

### Strengths and limitations of this study

The complementary outcomes we examined include not only validated rating scales but also participant-reported clinical diagnoses. This selection of measures, therefore, adds breadth compared to previous research in this area. Furthermore, there is limited published work on multimorbidity and polypharmacy in midlife, so this work fills an important gap.

The data available were collected in the baseline pilot phase of PREVENT Dementia, only permitting exploratory cross-sectional analyses of 210 participants. The cohort was designed as a longitudinal study and follow-up data collections are ongoing. Cross-sectional analysis leaves questions about direction of causality unclear. It is known, for example, that all mental disorders are associated with later physical health consequences, so the findings from this study may reflect reverse causality in that people who were originally depressed experienced physical health deterioration.^33^ The recruitment of volunteers who are likely to have an interest in dementia research limits the generalizability of our results. The sample is 89.5% Caucasian which is close to the UK proportion of 87.2% but less diverse than the population of London where 59.8% of people are white.^34^

Although the STAI and CES-D feature some questions on somatic symptoms of anxiety and depression, the majority are cognitive symptoms so this is unlikely to capture physical symptoms of physical conditions. However, people with anxiety and depression, particularly older people, can report physical symptoms as the primary complaint.^35,36^ This may lead to seeking medical attention and, therefore, receiving more diagnoses of physical conditions. The self-report nature of the PREVENT Dementia medical history and the overlap between the clinical presentations of depression and anxiety disorder meant that a number of participants reported both conditions. The questions in the screening tests mean that CES-D includes symptoms of generalized anxiety disorder and STAI, symptoms of depression.^22,37^ In addition, there is an overlap between multimorbidity and polypharmacy and we did not adjust for either when assessing each exposure.^38^

With such a sample size, groups within the data set can be small, for example, only 26 participants reported current use of antidepressant medication. There are also more women (148, 70.5%) than men in the sample, so when groups are subdivided by gender, they can become very small – for example, only seven men took antidepressants. It is important to recognize the role of chance in analyses on these numbers, and effect size could be over-estimated. In addition, we adjusted for covariates that were clinically relevant and statistically significant in preparatory analyses but there may be residual confounding from unmeasured factors. These exploratory analyses will inform future research in a larger sample from this cohort.

The nature of the PREVENT Dementia initial visit is that all the medical history and medications are self-reported. This can lead to several types of bias including recall bias and social desirability bias.^39^ Self-reported depression may be more sensitive than CES-D for identifying people with a clinical diagnosis who have received treatment and, therefore, perform better on testing than they might have done untreated. However, participants may also report depression that has not been clinically diagnosed, more so perhaps than a physical condition. Previous studies comparing self-report with diagnostic or screening tests for depression have remarked upon this complex relationship.^40^ Self-reported antidepressant use in cohort studies, however, has been found to correlate strongly with prescription records.^41^

In all but one analysis, an apparent association between exposure and outcomes ceased when including antidepressant use as a covariate. This implies that taking antidepressants, perhaps as a marker for mental disorders (fully or partially treated), is an important explanation in the pathway between chronic conditions, medication use and anxiety and depression. The overlap between physical and mental illness is complex and difficult to capture but we attempted to understand it by approaching it from several different angles. This is a strength over previous research, which has not attempted to account for the treatment of depression or anxiety.^5,7–9^ In addition, antidepressant use suggests a preexisting diagnosis of mental illness, but detailed temporality of mental and physical diagnoses cannot be ascertained in cross-sectional data. Future waves of the PREVENT Dementia study will allow longitudinal exploration of this issue.

### Implications

The presence of associations between increasing chronic conditions, medications and depression supports the important interaction of physical health and resulting medication burden with mental health, even in midlife. The modest nature of these results in a small sample size limits the certainty with which conclusions can be drawn but reinforces the need to corroborate them in larger data sets. A particular strength of completing this work in a pilot wave of an ongoing longitudinal study is the opportunity to revisit the analyses when data from future waves are available. In these cross-sectional analyses, we were unable to evaluate the implications for participants’ future development of dementia, but follow-up may allow this. The focus on midlife individuals may also inform strategies to improve health in later life. For example, if midlife physical health can be optimized, this may reduce later anxiety and depression.

## Conclusions

In this cross-sectional study of a middle-aged cohort of volunteers, we found associations between increasing chronic conditions and self-reported depression, depressive symptoms and self-reported anxiety disorder but not anxiety symptoms. In addition, there were associations between increasing number of medications and depression (both self-reported and according to a screening scale) but not anxiety. The use of antidepressants, as a marker for preexisting mental illness, attenuated the associations found. This work adds to understanding of physical and mental health multimorbidity.

## Supplemental material

Supplemental Material, STROBE_checklist_cross-sectional_20190625 - Associations between midlife chronic conditions and medication use with anxiety and depression: A cross-sectional analysis of the PREVENT Dementia study Click here for additional data file. Supplemental Material, STROBE_checklist_cross-sectional_20190625 for Associations between midlife chronic conditions and medication use with anxiety and depression: A cross-sectional analysis of the PREVENT Dementia study by Lucy E Stirland, Sarah Gregory, Tom C Russ, Craig W Ritchie and Graciela Muniz-Terrera in Journal of Comorbidity

## Appendix 1

**Table 1A.** List of included chronic physical conditions with prevalence in PREVENT Dementia baseline phase.

Condition	Definition	Prevalence, *n* (%)
Eye disease	Currently active	113 (53.8)
Asthma	Currently active	28 (13.3)
Migraine	Currently active	27 (12.9)
Sleep disorder	Currently active	25 (11.9)
Gastro-oesophageal reflux disease	Currently active	23 (11.0)
Hypertension	Currently active	22 (10.5)
Irritable bowel syndrome	Currently active	17 (8.1)
Other musculoskeletal condition (each free text entry checked for relevance)	Currently active or ever recorded	17 (8.1)
Cardiac arrhythmia	Currently active	14 (6.7)
Cancer	Ever diagnosed	14 (6.7)
Osteoarthritis	Currently active	26 (12.4)
Degenerative disc disease	Currently active	13 (6.2)
Anaemia	Currently active	12 (5.7)
Chronic constipation	Currently active	12 (5.7)
Hypothyroidism	Currently active	11 (5.2)
Diabetes	Currently active	7 (3.3)
Peripheral vascular disease – venous	Currently active	6 (2.9)
Other gastrointestinal disorder (each free text entry checked for relevance)	Currently active or ever recorded	5 (2.4)
Inflammatory bowel disease	Ever recorded	4 (1.9)
Other genitourinary/reproductive disorder (each free text entry checked for relevance)	Currently active or ever recorded	4 (1.9)
Other haematological disorder (each free text entry checked for relevance)	Currently active or ever recorded	3 (1.4)
Cholelithiasis	Currently active	3 (1.4)
Diverticulitis	Currently active	3 (1.4)
Peripheral nerve disorder	Currently active	3 (1.4)
Angina	Currently active	2 (1.0)
Other cardiovascular disease (each free text entry checked for relevance)	Currently active or ever recorded	2 (1.0)
Peptic ulcer disease	Currently active	2 (1.0)
Liver disease (excluding hepatitis)	Currently active	2 (1.0)
Other eye disease (each free text entry checked for relevance)	Currently active or ever recorded	2 (1.0)
Gout	Currently active	2 (1.0)
Stroke	Ever diagnosed	2 (1.0)
Other neurological disorder (each free text entry checked for relevance)	Currently active or ever recorded	2 (1.0)
Valvular heart disease	Currently active	1 (0.5)
Coronary artery disease	Ever diagnosed	1 (0.5)
Gastrointestinal bleed	Currently active	1 (0.5)
Benign prostatic hyperplasia	Currently active	1 (0.5)
Nephrolithiasis	Currently active	1 (0.5)
Hyperthyroidism	Currently active	1 (0.5)
Other metabolic (each free text entry checked for relevance)	Currently active or ever recorded	1 (0.5)
Immune deficiency	Currently active	1 (0.5)
Other immunological condition (each free text entry checked for relevance)	Currently active or ever recorded	1 (0.5)
Aortic aneurysm	Ever diagnosed	0
Cholecystitis	Currently active	0
Chronic obstructive pulmonary disease	Ever diagnosed	0
Collagen vascular disease	Currently active	0
Congenital heart disease	Currently active	0
Congestive heart failure	Ever diagnosed	0
Hepatitis	Currently active	0
Kidney disorder	Currently active	0
Pacemaker	Ever reported	0
Pancreatitis	Currently active	0
Parkinson’s disease	Ever diagnosed	0
Peripheral vascular disease – arterial	Currently active	0
Seizure/convulsion disorder	Currently active	0
Tuberculosis	Currently active	0

## Appendix 2

### Results of supplementary analyses stratified by gender

**Table 2A.** Women only.

		Exposure					
Outcome	**Model**	Chronic physical conditions			Medications excluding antidepressants		
		Coefficient (95% CI)	OR^a^ (95% CI)	*p* Value	Coefficient (95% CI)	OR^a^ (95% CI)	*p* Value
Depression							
CES-D	Model 1	**0.81 (0.12, 1.51)**		**0.021**	**0.74 (0.01, 1.48)**		**0.048**
	Model 2	0.62 (−0.09, 1.32)		0.085	0.50 (−0.25, 1.25)		0.190
Self-reported depression	Model 1		**1.43 (1.12, 1.84)**	**0.005**		**1.44 (1.12, 1.90)**	**0.005**
	Model 2		1.33 (0.88, 2.05)	0.177		1.25 (0.84, 2.06)	0.329
Anxiety							
STAI	Model 1	0.33 (−0.50, 1.16)		0.436	0.10 (−0.79, 0.98)		0.828
	Model 2	0.16 (−0.69, 1.02)		0.707	−0.12 (−1.03, 0.79)		0.795
Self-reported anxiety disorder	Model 1		**1.71 (1.34, 2.25)**	**<0.001**		1.25 (0.98, 1.58)	0.061
	Model 2		**1.72 (1.29, 2.40)**	**<0.001**		1.05 (0.79, 1.42)	0.732

OR: odds ratio; CI: confidence interval; CES-D: Center for Epidemiologic Studies Depression; STAI: State-Trait Anxiety Inventory.

^a^ OR per unit increase in number of chronic conditions or medications: model 1: women only (*n* = 148), adjusted for age; model 2: women only (*n* = 148), adjusted for age and use of antidepressants for psychiatric indication.

**Table 2B.** Men only.^a^

		Exposure				
Outcome	Model	Chronic physical conditions		Medications excluding antidepressants		
		Coefficient (95% CI)	*p* Value	Coefficient (95% CI)	*p* Value	
Depression						
CES-D	Model 1	0.37 (−1.01, 1.75)	0.592	**1.10 0.22, 1.97)**	**0.015**	
	Model 2	0.36 (−1.09, 1.80)	0.622	**1.10 (0.21, 2.00)**	**0.016**	
Anxiety						
STAI	Model 1	−0.59 −2.03, 0.84)	0.412	0.55 (−0.41, 1.50)	0.256	
	Model 2	−0.52 (−2.02, 0.99)	0.492	0.60 (−0.37, 1.57)	0.221	

CI: confidence interval; CES-D: Centre for Epidemiologic Studies Depression; STAI: State-Trait Anxiety Inventory; model 1: men only (*n* = 62), adjusted for age; model 2: men only (*n* = 62), adjusted for age and use of antidepressants for psychiatric indication.

^a^Self-reported depression and anxiety disorder not included owing to small number of male participants reporting these diagnoses.
